# Action Spectra of Zebrafish Cone Photoreceptors

**DOI:** 10.1371/journal.pone.0068540

**Published:** 2013-07-05

**Authors:** Duco Endeman, Lauw J. Klaassen, Maarten Kamermans

**Affiliations:** 1 The Netherlands Institute for Neuroscience, Department of Retinal Signal Processing, Amsterdam, The Netherlands; 2 Academic Medical Center, Department of Neurogenetics, Amsterdam, The Netherlands; University Zürich, Switzerland

## Abstract

Zebrafish is becoming an increasingly popular model in the field of visual neuroscience. Although the absorption spectra of its cone photopigments have been described, the cone action spectra were still unknown. In this study we report the action spectra of the four types of zebrafish cone photoreceptors, determined by measuring voltage responses upon light stimulation using whole cell patch clamp recordings. A generic template of photopigment absorption spectra was fit to the resulting action spectra in order to establish the maximum absorption wavelength, the A2-based photopigment contribution and the size of the β-wave of each cone-type. Although in general there is close correspondence between zebrafish cone action- and absorbance spectra, our data suggest that in the case of MWS- and LWS-cones there is appreciable contribution of A2-based photopigments and that the β-wave for these cones is smaller than expected based on the absorption spectra.

## Introduction

Zebrafish (*Danio rerio*) is a widely used model system in developmental studies. This animal is also attractive for the study of the visual system because they are vision-dependent predators exploiting a cone-dominated retina, which renders high visual acuity [1]. Presently zebrafish is the only vertebrate model system that relies profoundly on vision, which can easily be used in behavioral studies, of which the genome is known and is relatively easy genetically modifiable. Consequently, in recent years, there has been an increasing interest for this animal model in the field of visual neuroscience [2]–[4]. For any functional study in this field, it is essential to know the action spectra of the retinal photoreceptors since they form the input layer of the visual system. The current paper describes the action spectra of cone photoreceptors of the zebrafish retina.

The zebrafish retina contains four distinct spectral cone types: LWS (long wavelength sensitive), MWS (middle wavelength sensitive), SWS (short wavelength sensitive), and UVS (ultraviolet sensitive), which are evenly distributed across the retina in a regular mosaic. These spectral cone types overlap with four morphological cone subtypes: long (or principal) and short (or accessory) members of double cones (LWS- and MWS-cones, respectively), long-single cones (SWS-cones) and short-single cones (UVS-cones) [5]. However, it is becoming clear that the spectral sensitivity of the different cone types of zebrafish is not straightforward.

Photopigments, the proteins initiating light transduction in photoreceptors, are composed of an opsin and a chromophore. Zebrafish may express 9 different opsins, each coded by a separate gene [6], [7]. LWS-cones can express two opsins (LWS-1 and LWS-2), MWS-cones four opsins, (RH2-1, RH2-2, RH2-3, and RH2-4) and UVS- and SWS-cones only one opsin (SWS1 and SWS2, respectively). The opsin of rods is coded by a separate gene (RH1). These different opsins give rise to distinct absorbance spectra of photopigments. Their expression follows a spatiotemporal pattern, meaning that certain opsins are expressed at restricted locations of the retina at given developmental stages [8].

Additional variation in the spectral sensitivity of zebrafish cone types is induced by the fact that their photopigments can be constituted with either a vitamin A1- (retinal), or A2-based (3,4-didehydroretinal) chromophore. The absorbance spectra of photopigments constructed with vitamin A2-based chromophores, called porphyropsins, are shifted to longer wavelengths compared to their vitamin A1-based analogues, termed rhodopsins [9], not to be confused with the photopigments of rods. This shift increases with longer peak absorbance wavelengths of the A1-based photopigment [10]. By expressing different mixtures of vitamin A1- and A2-based photopigments, animals can tune the spectral sensitivity of their photoreceptors, e.g. to match the spectral content of environmental light [11]. Adult zebrafish have been shown to possess a fully functioning vitamin A1/A2 interchange system [12]. Whether the A1- and A2-based photopigment expression ratio is actively modified and under which conditions this occurs is unknown.

Previously, the absorbance spectra of the different zebrafish cone types have been investigated using microspectrophotometric (MSP) techniques [13]–[16]. This has also been done following exogenous thyroid hormone application, which induces expression of vitamin A2-based chromophore photopigments [12]. Accordingly, application of thyroid hormone shifted absorbance spectra peaks to longer wavelength. Furthermore, the absorbance spectra of isolated zebrafish photopigments reconstituted with an A1-based chromophore for all different cone opsin types have been determined [7].

Despite the abundance of knowledge concerning the absorbance spectra of the photopigments of zebrafish photoreceptors, the action spectra of zebrafish cones have not been described previously. The action spectra of photoreceptors of the closely related Giant danio (*Danio aequipinnatus*) have been described [17], but there is no a priori reason to assume that zebrafish will have similar action spectra. Action spectra are constructed based on electrophysiological recordings of photoreceptors and illustrate effectiveness of stimulus wavelengths in generating changes in membrane potential or current. The action spectra are not necessarily similar to the absorbance spectra of photopigments [18]. The action spectra of photoreceptors are needed for physiological studies of the zebrafish visual system because they determine the signal transmitted to subsequent neuronal layers of the retina.

In the present study, we have recorded voltage responses of zebrafish UVS-, SWS-, MWS-, and LWS-cones to light of different wavelengths. A generic template of the absorbance spectra of A1- and A2-based photopigments [2], [15], [19] was fit to the action spectra in order to calculate peak action wavelengths and the percentage of A1- and A2-based photopigment contribution.

For the most part our data agree with MSP-data regarding peak sensitivity wavelengths. However, MWS- and LWS-cones in our sample appeared to have substantial amounts of A2-based photopigments. Moreover, for the MWS- and LWS-cones, response amplitudes at shorter wavelengths (β-band) were significantly smaller than expected on the basis of the absorbance spectra.

## Methods

### Preparation

Wild type zebrafish, *Danio rerio*, (AB strains) were originally obtained from the Zebrafish International Resource Center (Eugene, OR, USA). Lines were maintained in our own facility. Male and female fish were housed in aquaria at 28° to 28.5°C under a 14/10 hours light/dark cycle.

Light adapted adult zebrafish (aged 1–2 years) were kept in the dark for at least 5 min to facilitate the isolation of the retina from the pigment epithelium. All further steps in the preparation were performed in the dark under dim red (λ = 650 nm) illumination. Fish were euthanized by immersion in ice water and decapitated. The head was bisected along the anterior/posterior axis, and the eyes were removed and hemisected. Subsequently, retinas were adhered to a small piece of tissue paper, which was placed receptor side up in a recording chamber and continuously superfused (1.5 ml/min) with oxygenated Ringer’s solution (pH 7.8, 20°C).

The recording chamber was mounted on a microscope (model Eclipse E600-FN, Nikon, Tokyo, Japan) equipped with infrared (λ >800 nm; wratten filter 87c, Kodak, Rochester, NY, U.S.A.) differential interference contrast optics. The preparation was viewed on an LCD monitor by means of a 60× waterimmersion objective (N.A. 1.0) and a CCD camera (Philips, Eindhoven, The Netherlands). Recordings started at least 10 minutes after mounting the recording chamber on the microscope.

### Solutions

The Ringer’s solution contained (in mM) 102.0 NaCl, 2.6 KCl, 1.0 MgCl_2_, 1.0 CaCl_2_, 28.0 NaHCO_3_, 5.0 glucose and was continuously gassed with 2.5% CO_2_ and 97.5% O_2_ yielding a pH of 7.8.

The pipette medium contained (in mM) 10 KCl, 96 KGluconate, 1 MgCl_2_, 0.1 CaCl_2_, 5 EGTA, 5 HEPES, 5 ATP-Na_2_, 1 GTP-Na_3_, 0.2 3′: 5′ -cGMP-Na, 20 Phosphocreatine-Na_2_, 50 units/ml creatine phosphokinase. The pH of the pipette medium was adjusted to 7.2 with KOH. The liquid junction potential was calculated (after Barry & Lynch [20] and Ng & Barry [21]) and the voltage value was adjusted accordingly. All chemicals were supplied by Sigma (Zwijndrecht, The Netherlands).

### Electrodes and Recording Equipment

Patch-pipettes were pulled from borosilicate glass capillaries (GC150TF-10, Harvard Apparatus Ltd., Kent, United Kingdom.) with a Brown-Flaming micropipette puller (P-87, Sutter Instruments Company, Novato, CA, U.S.A.) and had resistances between 5 and 8 MΩ when filled with pipette solution and measured in Ringer’s solution. The electrodes were placed in a PCS-5000 patch clamp micromanipulator (Burleigh Instruments Inc., Fishers, NY, U.S.A.) and connected to an Axopatch 200 Patch Clamp Amplifier (Axon Instruments Inc., Union City, CA, U.S.A.). Data acquisition and control of the optical stimulator were made by means of a CED 1401 AD/DA converter (Cambridge Electronic Design Ltd., Cambridge, United Kingdom), a Windows-based computer system and Signal (Cambridge Electronic Design Ltd., Cambridge, United Kingdom). Recordings were performed in current-clamp mode, with the holding current kept at 0 pA. Data were sampled at 1 kHz for all stimulus protocols and filtered at 1 kHz using a four-pole Bessel filter.

### Optical Stimulator

The light stimuli were generated with a 450-W xenon arc (Osram, Munich, Germany). Before reaching the retina, the light passed through a series of neutral density filters (Schott, Mainz, Germany), interference filters (Melles Griot, Zevenaar, The Netherlands) with peak transmissions at 380, 400, 450, 500, 550, 600 nm and a 8 nm bandwidth, and circular neutral density wedges (Barr & Stroud, Glasgow, United Kingdom). Light stimuli were projected onto the retina through the objective by means of mirrors and lenses, which resulted in a spot with a diameter of 65 µm. The light intensity at the focal plane of the microscope was measured with a radiometer (model 50–245, irradiance head J1812, Tektronix, Bracknell, United Kingdom). Throughout the paper, a photon flux density of 1.0 * 10^9^ photons m^−2^ sec^−1^ corresponds to an intensity of 0 log.

### Recording Procedure

To determine the spectral sensitivity of cones, whole-cell recordings were made while the photoreceptors were stimulated with 500 ms light flashes of various wavelengths and intensities. The interstimulus interval was always more than 3 seconds. For each wavelength, the mean amplitude of the sustained light response between 250 and 500 ms after light onset was plotted as a function of the stimulus intensity. Using the least-square method, a Hill relation (eqn. (1)) was fit globally through these data points:
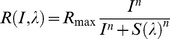
(1)where: 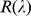 is the response (in mV) as function of stimulus wavelength (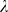), 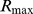 is the maximal response amplitude (in mV), 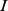 is stimulus intensity, 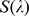 is the stimulus intensity needed for half maximal response for a specific wavelength, and 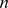 is a slope factor. 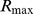 and 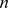 were shared parameters for the various intensity response curves for individual cells. This procedure resulted in one value for *R_max_* and one value for *n* per cell. Relative sensitivity is defined as the difference between 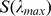 and 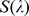, where 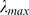 is the wavelength for which the photoreceptor is most sensitive. Absolute sensitivity (S_abs_) is equal to 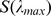. Based on wavelength sensitivity cones were classified as UVS-, SWS-, MWS-, or LWS-cones and grouped accordingly. Relative spectral sensitivity data are presented as mean ± *SD* and parameter differences were tested for significance (*p*<0.05) using a one-way ANOVA.

### Visual Pigment Templates

A generalized template, consisting of mathematical descriptions of the *α-* and *β*-band, for absorbance spectra of A1- and A2-based photopigments [15] was fit to the action spectra of cones. Following Govardovskii’s template, the *α*-band was defined by.
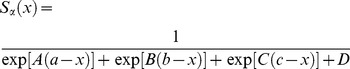
(2)

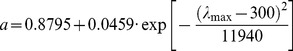
(3)for A1-based photopigments. For A2-based photopigments, the following set of parameters was used: 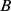 = 20.85, 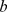 = 0.9101, 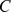 = −10.37, 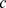 = 1.1123, 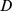 = 0.5343,
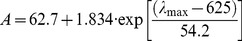
(4)and



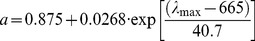
(5)The *β*-band was taken as the absorbance spectrum remaining after subtraction of the *α*-band, which was fit with a Gaussian:
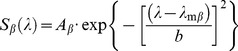
(6)


In the case of A1-based photopigments for fitting of the absorption spectra, 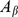 was 0.26,
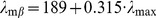
(7)and



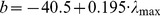
(8)For A2-based photopigments for fitting of the absorption spectra, 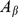 was 0.37,
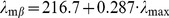
(9)and



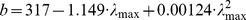
(10)The photopigment template was fit to cone data over a stimulus range which corresponded to a minimum relative sensitivity of −3 log units. Fits were evaluated by least square methods, with 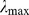 of the A1-based photopigment, the percentage of A1- and A2-based photopigment contribution and the percentage of *β*-band presence as free parameters. The 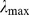 of MWS- and LWS-cone A2-based photopigments was determined by the following relation [10]:
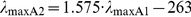
(11)


Fitting parameters are presented as mean ± *SD*. The A1- and A2-based photopigment contribution and *β*-band presence were tested for significance (*p*<0.05) using a one-tailed Student’s t-test.

## Results

### Response Properties of Zebrafish Cones

In order to determine the action spectra of zebrafish cone photoreceptors we measured light responses, using whole-cell current clamp, in isolated retinae. In total we successfully recorded from 3 UVS-, 3 SWS-, 6 MWS-, and 6 LWS-cones.

**Figure 1:**
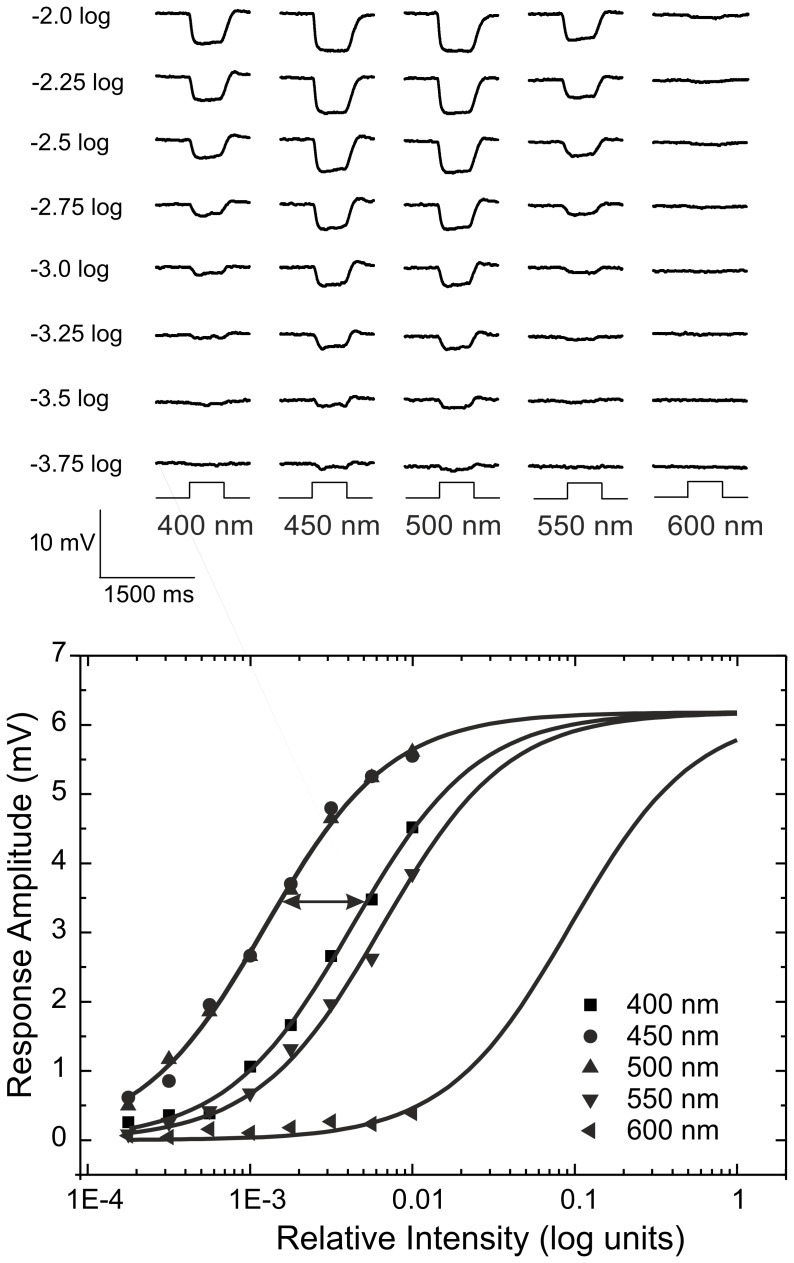
Examples of experimental data and analysis. The top panel displays voltage responses of an MWS-cone to 500 ms light stimuli. The intensity of the stimulus decreases from top to bottom as indicated by the relative intensity to the left of the traces. The stimulus wavelength increases from left to right as indicated below the traces. The bottom panel illustrates how cone responses were converted to spectral sensitivity functions using data from the same MWS-cone. Response amplitudes for different stimulus wavelengths were plotted against stimulus intensity. Subsequently Hill-relations were fit through data for each stimulus wavelength and the difference in half-maximal activation relative to the wavelength for which the cone was most sensitive (double arrow) was determined.

In Fig. 1 (Top) light responses of an MWS-cone to 500 ms light flashes of varying wavelengths and intensities are shown. The cone had a resting membrane potential of −39.4 mV and a maximal sustained response amplitude of 6.2 mV.

Intensity response curves for the various stimulus wavelengths were constructed by plotting the sustained response amplitudes as function of stimulus intensity (Fig. 1, bottom), and subsequently fitting Hill relations (solid lines, see Methods section for details) through the data points. Average parameter values of the Hill relations (n and S_abs_), mean resting membrane potentials (V_rest_) and mean maximum response amplitudes (R_max_) for the various cone types are given in Table 1. None of these values differed significantly between cone types.

**Table 1:** Properties of zebrafish cones.

Cone-type	V_rest_ (mV)	R_max_ (mV)	N	S_abs_ (log)
UVS-cone (*n* = 3)	−40.6±4.8	12.0±7.4	1.1±0.2	2.8±0.3
SWS-cone (*n* = 3)	−39.7±3.5	6.5±1.7	1.1±0.3	2.7±0.2
MWS-cone (*n* = 6)	−42.3±2.4	12.7±6.1	1.2±0.1	3.1±0.2
LWS-cone (*n* = 6)	−35.6±3.7	11.5±4.7	1.3±0.2	2.9±0.2
All cones (*n* = 18)	−36.7±4.2	11.1±5.5	1.2±0.2	2.9±0.3

Average properties of the various zebrafish cones types. V_rest_, resting membrane potential; R_max_, maximum response amplitude relative to V_rest_; n, coefficient of fit Hill-relation; S_abs_ absolute sensitivity (see Methods section for details). Parameters are presented as mean ± *SD.*

### Spectral Sensitivity Functions of Zebrafish Cones

**Figure 2:**
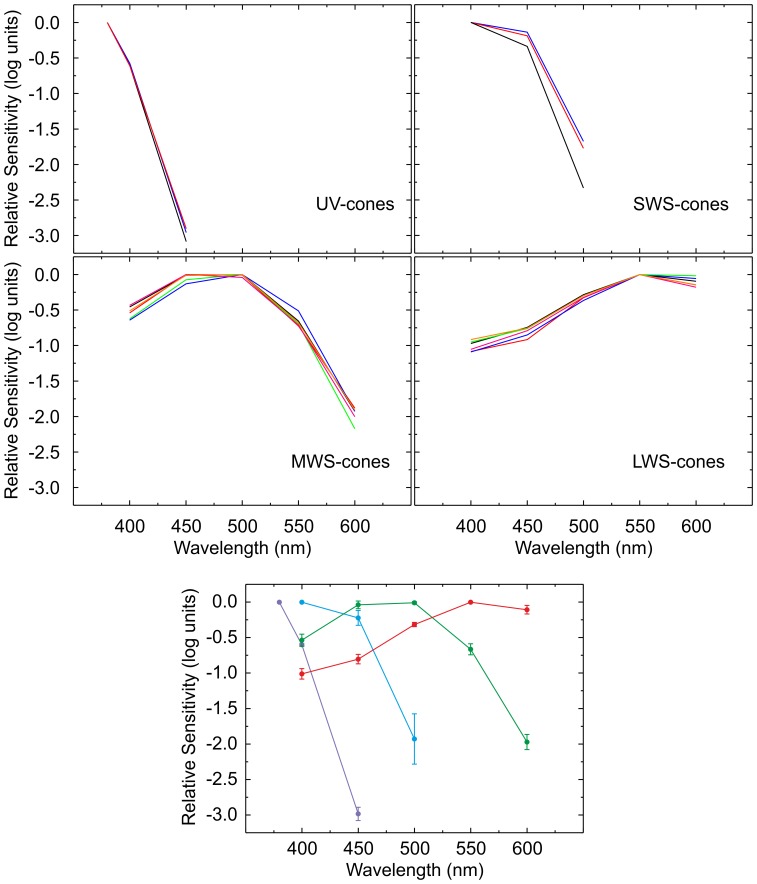
Action spectra of individual cells and averages per cone-type. In the top four panels the relative sensitivity is plotted against the stimulus wavelength of individual cones grouped according to cone-type as indicated in the graph. The bottom panel displays the average spectral sensitivity for the different cone-types.

To construct spectral sensitivity functions of cones, we determined for each stimulus wavelength the intensity needed to obtain the half maximal response amplitude. We measured voltage responses for 8 light intensies per wavelength, derived an intensity response relation and fit a Hill equation through the data points. ΔS(λ) was determined relative to S(λ) of the most sensitive wavelength (double arrow, Fig. 1, bottom). This value was defined as the relative sensitivity for that specific stimulus wavelength. These values were plotted against the corresponding stimulus wavelengths for individual cells in Fig. 2A, B, C and D. In Fig. 2E the mean spectral sensitivity of UVS-cones, SWS-cones, MWS-cones and LWS-cones are displayed in ensemble.

In order to determine the peak wavelength of the action spectrum and A1- vs. A2-based photopigment contribution, we fit a photopigment template [15] to data from each recorded cone individually. The template mathematically describes the shape of the absorbance spectrum of visual pigments. It consists of a main absorbance band, the α-band, and a secondary absorbance band, the β-band, which is dominant at shorter wavelengths. During the fitting procedure, optimal values for the peak absorbance wavelength of the A1-based photopigment, the percentage of A1- and A2-based photopigments contribution and the percentage of the β-wave presence relative to the absorption spectra templates were calculated. All other parameters were set to generic values (see Methods section for details). The parameters of fitting the Govardovskii template to the data are listed in table 2.

**Table 2:** Parameters of photopigment template fits.

	UVS-cone	SWS-cone	MWS-cone	LWS-cone
λ_max_ (nm)	365±2	416±5	483±1	574±9
λ_max_ A1 (nm)	365±2	416±5	480±1	556±5
λ_max_ A2 (nm)	–	–	492±1	612±9
Presence A1 (%)	–	–	71±10	68±19
Presence β-band (%)	–	–	49±41	31±6

Mean parameter values for the various cone types. Parameters are presented as mean ± *SD.*

**Figure 3:**
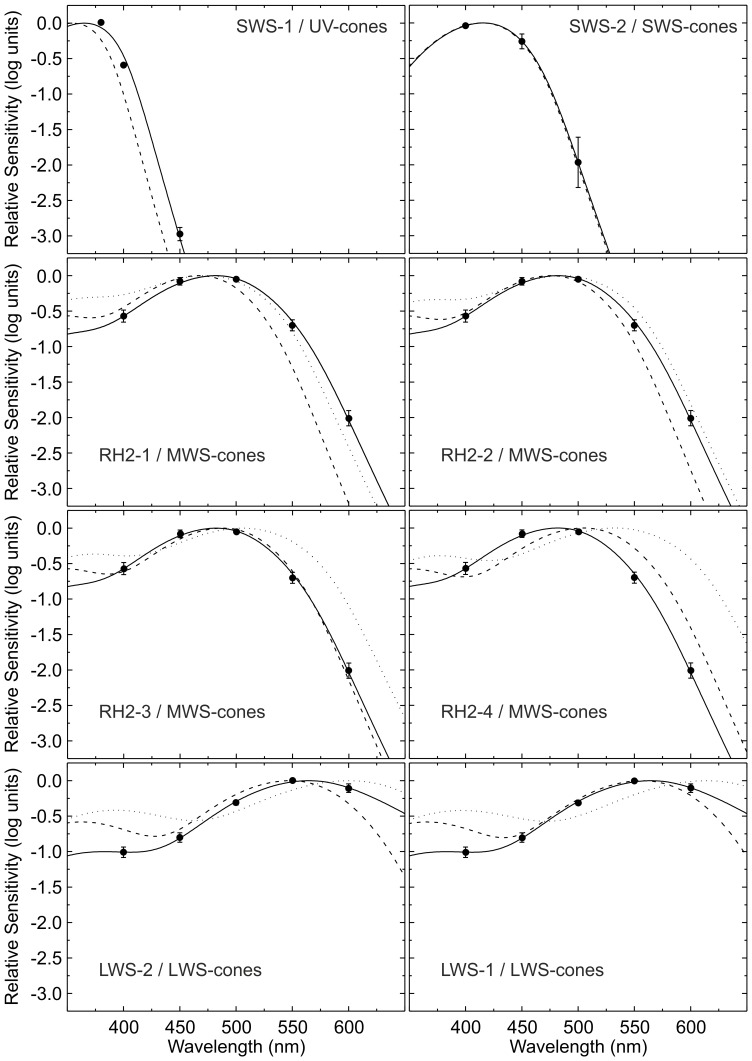
Fits of pigment template to experimental data. This figure displays the fits (solid lines) of the photopigment template [15] to the average experimental data per cone-type with the peak wavelength of the A1-based photopigment, the ratio between A1- and A2-based photopigment and the presence of the β-wave relative to the original photopigment template as free parameters. For comparison the spectral sensitivity functions of corresponding A1- (dashed lines) and A2-based (dotted lines) photopigments are also plotted. These were constructed according to the generic photopigment template in combination with their peak absorbance wavelength as measured *in vitro* [7]. The UV-cone action spectra deviates considerable from the UV template, presumably due to the small number of data points (top row, left). The action spectrum of the SWS cones was fully overlapped the SWS-2 adsorption spectrum (top row, right). For the MWS cones, both RH2–2 and RH2–3 templates were covering the MWS action spectrum, while RH2–1 and RH2–4 templates could not describe the action spectrum properly. Finally both LWS-1 and LWS-2 templates covered the LWS action spectrum.

Fig. 3 illustrates how the cone action spectra relate to the absorbance spectra of cone photopigments. In this figure the fits of mean data per cone-type are plotted along with the absorbance spectra of photopigments, according to the Govardovskii template. The latter are subdivided into opsins expressed in corresponding cone-types in combination with either an A1- or A2-based chromophore.

Zebrafish UVS-cones express a single opsin, SWS1, which has a peak absorbance wavelength of 355 nm, when A1-based (Chinen, Hamaoka, Yamada, & Kawamura, 2003). For these cones the β-band is outside the stimulation range (380–600 nm) and the Govardovskii template has not been evaluated for its A2-based photopigment. Therefore, UVS-cones were fit with 0% β-band and 0% A2-based photopigment contribution. With these constraints the mean peak wavelength of the UVS-cone action spectrum was calculated to be 365±2 nm (*n* = 3).

Similar conditions apply to SWS-cones, which also express a single opsin, SWS2. Its A1-based photopigment has a peak absorbance wavelength of 415 nm (Chinen, Hamaoka, Yamada, & Kawamura, 2003), almost equal to the mean peak wavelength calculated for the action spectrum of SWS-cones, 416±5 nm (*n* = 3).

MWS-cones express one of four opsins, RH2-1, RH2-2, RH2-3 or RH2-4, which, in combination with an A1-based chromophore, have peak absorbance wavelengths of 467, 476, 488 and 505 nm, respectively (Chinen, Hamaoka, Yamada, & Kawamura, 2003). There was little variation in the peak wavelength of individual fits of MWS-cone action spectra, which was 483±1 nm (*n* = 6) on average. Greater amount of variation was present in the A1:A2 based photopigment ratio. On average this was 71∶ 29±10% (*n* = 6), which differed significantly from 100% A1- based photopigment presence (p<0.01). The relative amplitude of the β-band was highly variable. On average 49±41% (*n* = 6, p = 0.03) of the β-band was present relative to the original photopigment template. This corresponds to a β-band peak amplitude which is 14±12% of that of the α-band.

LWS-cones can express either LWS-1 or −2 opsin. Their A1-based photopigments have peak absorbance wavelengths of 558 and 548 nm, respectively (Chinen, Hamaoka, Yamada, & Kawamura, 2003). We found some variation in the peak wavelength of LWS-cone action spectra. On average λ_max_ was 574±9 nm (*n* = 6). Similar to MWS-cones, this wavelength was partly achieved by the presence of A2-based photopigments. The A1:A2 based photopigment contribution was variable as well. On average it was best fit by 68∶ 32±19% (*n* = 6), which differed significantly from 100% A1- based photopigment presence (n = 6, p<0.01). There was less variation in the percentage of β-band presence for LWS-cones. On average 31±6% (*n* = 6, p<0.01) of the β-band was present relative to the standard photopigment template, corresponding to a β-band peak amplitude which is 9.1±1.6% of that of the α-band.

## Discussion

In this study we have determined the action spectra of LWS-, MWS-, SWS- and UVS-cones of the adult zebrafish retina, by measuring their responses to different wavelengths of light and subsequently fitting a template of photopigment absorbance spectra to the results. Thus we calculated peak absorbance wavelengths, the ratio of A1- and A2-based photopigments and the size of the β-band compared to the photopigment template. Spectral sensitivity obtained by our electrophysiological measurements generally agreed with the data obtained by MSP in adult zebrafish (see table 3; [5], [12]–[14], [16]and with the absorbance spectra measured *in vitro* [7] (see table 3). Furthermore, they are comparable with the action spectra determined for the giant danio (*Danio aequipinnatus*) [17]. However, we found also some distinct differences between the absorption and action spectra which are discussed below.

**Table 3:** Comparison of zebrafish cone spectral sensitivity data.

Opsin	*In vitro*	Cone-type	*In situ* (MSP)					Current study
SWS-1	354.6±0.5*^a^*	UVS-cone		362±3*^c^*		361*^e^*	361±3*^f^*	365±2
SWS-2	416.0±1.0*^a^*	SWS-cone	417±5*^b^*	415*^c^*	407±2*^d^*	414*^e^*	411±5*^f^*	416±5
RH2-1	466.5±1.5*^a^*	MWS-cone	478±9*^b^*	480*^c^*	473±5*^d^*	483*^e^*	482±6*^f^*	483±1
RH2-2	475.7±0.5*^a^*							
RH2-3	488.0±0.0*^a^*							
RH2-4	504.9±0.7*^a^*							
LWS-1	557.7±3.3*^a^*	LWS-cone	556±6*^b^*	570*^c^*	564±6*^d^*	567*^e^*	565±10*^f^*	574±9
LWS-2	548.3±0.5*^a^*							

λ_max_ values (in nm) of zebrafish A1-based photopigments and cone-types from literature. Parameters are presented as mean ± *SD*. *^a^* Chinen et al. (2003); *^b^* Nawrocki et al. (1985); *^c^* Robinson et al. (1993); *^d^* Cameron (2002); *^e^* Govardovskii et al. (2000); *^f^* Allison et al. (2004).

### Measuring Action Spectra in the Whole Mounted Retina

The reported data were recorded from cone photoreceptors in whole mounted retinae. Given the intactness of this preparation, measured action spectra might in principle be influenced by heterologous coupling of cone photoreceptors and feedback received from horizontal cells. However, patch-clamp recordings of cone photoreceptors do not show any broadening of the action spectrum one would expect as a result of heterogeneous coupling but rather reflect a single cone type spectrum for all recorded cones. Also, recordings were made by patching the inner segment of cone photoreceptors, whereas the locus of coupling between cones usually is at the level of the cone pedicle by means of teleodendria [22], [23]. Furthermore the effect of horizontal cell feedback should be negligible since we used a relatively small spot to stimulate cones. This would only cause little polarization of horizontal cells. Moreover, the resultant of horizontal cell feedback can generally only be appreciated in photoreceptors by saturating direct light responses, since these are large compared to the current changes induced by horizontal cell feedback [24]. Therefore the obtained results reflect pure cone action spectra.

### Peak Sensitivity Wavelengths

UVS-cones express only a single opsin (SWS-1). Its A1-based photopigment has a peak absorbance wavelength around 355 nm (Chinen, Hamaoka, Yamada, & Kawamura, 2003). All MSP studies (see Table 3) find a somewhat higher value for the absorption spectrum of UVS-cones. The action spectrum of these cones reported in this paper is best fit with a peak wavelength of 365±2 nm. However the accuracy of this value is hampered because it fell outside the range of our stimulation wavelengths and the amount of reliable data points was limited for UVS-cones. Nevertheless the peak wavelength found for the action spectrum of UVS-cones is comparable to previous reports.

Like UVS-cones, SWS-cones express a single opsin (SWS-2), which has an A1-based peak absorbance wavelength of 416 nm (Chinen, Hamaoka, Yamada, & Kawamura, 2003). Most MSP (see Table 3) studies find a comparable value for the peak absorbance of SWS-cones. Likewise, we find a peak wavelength for the SWS-cone action spectrum around 416±5 nm.

Construction of action and absorbance spectrum for MWS-cones is more complicated than those of UVS- and SWS-cones, since they can express four types of opsins (RH2-1, −2, −3 and −4), with different A1-based peak spectral sensitivity wavelengths (467, 476, 488 and 505 nm, respectively (Chinen, Hamaoka, Yamada, & Kawamura, 2003)). Grouping of these cones can therefore lead to a heterogeneous pool of similar cone-types expressing different opsin-types. We have attempted to test this possibility by fitting the photopigment template to data from individual cones and comparing calculated values of the A1-based photopigment peak wavelength. The action spectra of all recorded cones were best fit when the peak wavelength was set around 480 nm with little variation, suggesting that they expressed the same type of opsin, presumably RH2-2. This opsin is also most abundantly expressed in MWS-cones of the adult zebrafish according to RT-PCR studies [7]. Due to the predicted presence of A2-based photopigments in MWS-cones the optimal value for its peak sensitivity wavelength was somewhat longer, 483 nm on average. This is in accordance with the peak absorbance wavelength of MWS-cones in most MSP studies (see Table 3).

LWS-cones can express two opsins, namely LWS-1 and LWS-2, with 558 and 548 nm A1-based peak absorbance wavelengths, respectively (Chinen, Hamaoka, Yamada, & Kawamura, 2003). The calculated peak wavelength of the A1-based photopigment between individual LWS-cone action spectra shows greater variation than in the case of MWS-cones and ranges from 550 to 564 nm. Therefore it is more difficult to ascribe an opsin-type to individual LWS-cones. However, the average calculated A1-based photopigment peak wavelength is 556±5 nm, near that of LWS-1. This is also the opsin most abundantly expressed in LWS-cones of the adult zebrafish according to the previously mentioned RT-PCR studies [7]. As in MWS-cones, recorded LWS-cones are predicted to have A2-based photopigment contribution and its peak sensitivity wavelength is larger than that of A1-based photopigments, namely 574±9 nm on average. This value is somewhat longer than the values obtained from MSP-studies (see Table 3).

### A2-based Photopigment Presence

Substitution of a vitamin A2-based chromophore for an A1-based one produces a red shift in the absorbance spectrum of a photopigment. The consequence is that the A2-using animals are more red-sensitive. By mixing A1-based and A2-based photopigments animals can tune their spectral sensitivity. Such observations have been done in other fish [9], [25], [26]. It seems that this phenomenon is dependent on environmental factors such as temperature, light and season. The suggestion is that animals shift between and mix chromophores dependent on the spectral composition of light in their environment. Using different mixtures of A1-, and A2-based chromophores, this tuning can be made dynamic in a single individual. For instance, eels change this ratio during migration to adapt to different light conditions [9].

We have estimated percentages for the presence of A1- and A2-based photopigments by linearly mixing of their absorbance spectrum templates. However the presence of A2-based photopigment has not been estimated for UVS- and SWS-cones because the photopigment template has not been evaluated for A2-based photopigments with peak wavelengths shorter than 440 nm.

In the case of MWS-cones, all individual fits predict the presence of A2-based photopigments, 29% on average, with some variation. Since the peak sensitivity wavelength of the action spectrum is comparable to most MSP data from literature this could suggest that in these studies there is also some A2-based photopigment present in native MWS-cones.

The same suggestion can be made for LWS-cones, for which most MSP studies, on average, find peak absorbance wavelengths longer than that of its A1-based photopigments. Correspondingly, the peak sensitivity wavelength of the LWS-cone action spectra reported here is partly established by the presence of on average 32% A2-based photopigments, which is about equal to the amount in MWS-cones, although with greater variation.

Changes in the expression of A1- and A2-based photopigments have been reported to occur in zebrafish rods due to temperature [27], but not in cones [12], nor to spectral rearing conditions of larvae [28]. However, adult zebrafish do possess a fully functioning A1/A2 interchange system [12]. It is tempting to hypothesize that the presence of A2-based photopigment in zebrafish cones as found here is related to the light conditions of our zebrafish facility. Yet the influence of the spectral content of light conditions on the spectral sensitivity functions of photoreceptors in adult zebrafish has not yet been tested.

### Size of the β-band

The action spectra of MWS- and LWS-cones show considerable deviation from the photopigment absorbance spectrum at the location of the β-band. We quantified this deviation by implementing a fitting parameter which set the size of the β-band relative to the photopigment template. There is large variation in its optimal value between individual fits and on average it is 49% for MWS-cones and 31% for LWS-cones corresponding to a β-band peak amplitude which is 14 and 9.1% of that of the α-band respectively.

The smaller amplitude of the β-band of action spectra relative to absorbance spectra has previously been described in photoreceptors of goldfish [29] and carp [30]. Since in the present study stimuli were projected from the photoreceptor side, there was no short wavelength filtering by structures in front of photoreceptor outer segments. It might be that the absorption of short wavelength photons by MWS- and LWS-cones as found in MSP studies leads to a conformational change in their photopigment, which is less effective in modulating the phototransduction cascade. Hence, it is not found in their action spectra. However, interpretation of results regarding the β-band is complicated by the fact that its mathematical description by the photopigment template is not as accurate as that for the α-band [15].

Previously, the action spectra of cone photoreceptors of giant danio (*Danio aequipinnatus*), a species closely related to zebrafish, have been reported [17]. The peak sensitivity wavelengths found were comparable to absorbance spectra previously described for zebrafish and the action spectra reported in the current study (as listed in Table 3). Using an alternative photopigment template, the action spectra of giant danio display less sensitivity in β-band region than predicted by the generic template based on photon absorbance by photopigments we used, in the case of LWS-cones. For MWS cones the size of the β-band was comparable to its generic value in the template we used in this study. However Palacios et al (1996) fitted these parameters using a linear scale, which diminishes differences between fit and experimental data at lower sensitivities. Therefore discrepancies between these results might be a consequence of the scales used during the fitting procedure or caused by implementation of different photopgiment templates.

### Conclusions

Action spectra of zebrafish cone photoreceptors correspond with the previously reported action spectra of related photopigments over the greater part of the used stimulation range (400–600 nm). However, sensitivity of MWS- and LWS-cones at shorter wavelengths is lower than expected based on the absorption spectrum of their pigments. Using a photopigment template we identified the opsins expressed and showed the presence of A2-based photopigments in our sample for these cone-types.
